# Exploring the Challenges of Afghan Refugee Women Facing COVID-19: A Qualitative Study in Iran

**DOI:** 10.3389/fpubh.2022.838965

**Published:** 2022-03-31

**Authors:** Javad Yoosefi Lebni, Halime Enayat, Seyed Fahim Irandoost, Ali Akbar Dehghan

**Affiliations:** ^1^Social Development and Health Promotion Research Center, Health Institute, Kermanshah University of Medical Sciences, Kermanshah, Iran; ^2^Department of Sociology, Shiraz University, Shiraz, Iran; ^3^Social Determinants of Health Research Center, Clinical Research Institute, Urmia University of Medical Sciences, Urmia, Iran

**Keywords:** women, Afghans, refugees, challenges, COVID-19, qualitative study

## Abstract

**Objective:**

Afghan refugee women in Iran confront many problems in dealing with COVID-19 due to their fragile conditions. Therefore, the aim of this study was to explore the challenges of Afghan refugee women in the face of COVID-19 in Iran with a qualitative approach.

**Methods:**

The present study was conducted with a qualitative approach among Afghan refugee women in Iran. Data were collected through semi-structured face-to-face and telephone interviews and were saturated with 30 women. Both targeted and snowball sampling were used. Data were analyzed using conventional qualitative content analysis and Graneheim and Lundman method. Guba and Lincoln criteria were observed to evaluate the quality of research results.

**Results:**

143 primary codes, 12 subcategories and five main categories were obtained from data analysis. The main categories include little knowledge and information (limited access to information resources, incomplete knowledge about COVID-19), family challenges (intensified experience of violence and conflict in the family, problems related to childbirth and pregnancy), socio-economic challenges (exacerbation of economic problems, high-risk living conditions, social isolation, limited support of social and health organizations), health issues (problems related to treatment, injustice in providing services and facilities) and problems after the death of a COVID-19 patient (burial challenges for immigrants; lack of funeral rites).

**Conclusion:**

Afghan refugee women in Iran are very vulnerable facing COVID-19 due to their fragile conditions. Social and health institutions and organizations need to provide more support to these women so that they can protect their health and that of their families against COVID-19 and the damage caused by it.

## Introduction

The outbreak of COVID-19 began in Wuhan, China (1, 2) and became the greatest threat to public health and the deadliest epidemic since 1918 (3, 4). COVID-19 has affected many people around the world so far, and the number of people with the disease and its mortality is increasing rapidly worldwide (5–7). As of February 02, 2022, the total number of people with COVID-19 has exceeded 383 million and the number of deaths has exceeded 5.7 million. The United States, India, and Brazil have the highest rates, respectively. The largest death rate belongs to the United States with more than 913,000 death cases. Iran has also registered more than 6,400,000 infected and over 132,000 death cases^1^.

High rates of infection and death and the rapid spread of COVID-19 have affected the economic, political and social dimensions of countries around the world (8–10) and have become a global crisis and the most important health issue (8, 11). This new virus has different consequences in different aspects of life; Low mental health (8, 12), suicide (13), lifestyle changes (14) and sleep disorders (15) have been reported as some consequences of the COVID-19 crisis.

Despite the widespread effects that COVID-19 has had on people's lives, these effects do not appear to be the same for everyone (16, 17). In other words, one of the groups at risk in the event of natural disasters and social crises are women, who are more affected by the limited access to resources, social and cultural restrictions, and the intensification of inequalities that already exist in society. They confront problems more than men do (18–20). Increased domestic violence (21, 22), family tension (23), fear (22, 23) Stress (24, 25), depression and anxiety (25, 26) have been reported as experiences of women during the COVID-19 outbreak.

Immigrants are also among those most affected by COVID-19 and the fragile situation of refugees at the time of COVID-19 is a global concern (27, 28). COVID-19 is a new challenge for refugees that can lead to increase anxiety, stress, depression, drug use, and violence against women and children due to fears of losing family members and limited access to health care (29). Physical distance results in feeling of social separation, which can lead to loneliness and emotional separation; it can disrupt social and economic life, and violate the rights of immigrants (30, 31). Rees and Fisher, 2020 showed that COVID-19 and its health and social consequences may provoke immigrants' past traumatic reactions, exacerbate mental health issues, and impair their functioning (32). A qualitative study of Syrian refugees in Lebanon found that refugees facing COVID-19 had issues such as having informal and unsanitary residences, insufficient water supply, limited use of masks, and limited access to health care (33).

Although the two groups of women and immigrants generally face many problems during the outbreak period, the challenges of immigrant women are broader in relation to COVID-19 (34). Mutambara et al. in a study among immigrant women in South Africa showed COVID-19 has exacerbated structural violence and insecurity for women, and if left unchecked by governments, the consequences of the disease will be long-term and more destructive (35). Most immigrants in Iran are Afghans, whose population has increased in the last 40 years due to cultural, religious, and linguistic commonalities with Iranians (36). One of the challenges for Afghan immigrants in Iran is the lack of insurance and the consequent high medical costs. Many of them do not have insurance due to not having a residence card, which leads to not going to medical centers and not wanting to be hospitalized and receive treatment and services. Also, access to disinfectants and prophylactics such as masks is low among Afghan immigrants to cope with COVID-19 (37). In addition to these challenges, Afghan immigrant women and girls in Iran are more disadvantaged than men during the COVID-19 outbreak because of the relative increase in physical and psychological vulnerability of refugee women during epidemics (38).

Afghan immigrant women have a special position in the family, and with the outbreak of COVID-19 and its consequences, due to their political and social restrictions in society, their roles and responsibilities may change and they may face new challenges. Therefore, it seems necessary to examine their experiences and problems during the outbreak of COVID-19. Also, no independent study (quantitative or qualitative) has been conducted in this field and the published contents are letters to the editor (37). Therefore, a qualitative study can better identify the challenges facing refugee women and take action to reduce these challenges. Thus, the aim of this study was to explore the challenges before Afghan refugee women in facing COVID-19 in Iran with a qualitative approach.

## Methods

In this study, conventional content analysis method was used. The study was conducted in Tehran, the capital of Iran that hosts a large number of Afghan refugees. Participants in the study were Afghan women living in Tehran. Inclusion criteria included being Afghan, living in Tehran and willingness to participate in the study. Exclusion criteria included interruption of the interview and failure to complete the process of answering the interview questions.

In order to reach the participants, targeted and snowball sampling were used. Thus, by referring to the service centers belonging to Afghan refugees and getting help from social facilitators, 12 Afghan women who were eligible for the study were selected. Then at the end of the interview. They were asked to introduce other women who were eligible to enter the study, and 18 other women were selected.

Semi-structured interviews were used to collect data, with 22 face-to-face interviews and eight telephone interviews. Of course, during the interview, notes were taken whenever necessary. In order for the participants to be able to easily share their experiences with the researcher, a trained woman with a master's degree in sociology and familiar with qualitative research was used to help researchers conduct the interviews.

At the beginning of the interview, the researcher introduced themselves and presented a brief resume, then explained the objectives of the research and its importance and necessity to the participants, and the interviews began with obtaining written consent from them. At first, some demographic questions such as age and education were asked, then interview continued with general questions such as “after the COVID-19 outbreak in Iran, what problems did you face?”, then more detailed questions were asked. A guide to interview questions is reported in Table 1. It should be noted that the guide to the interview questions was compiled and finalized after several sessions of discussion and exchange of views between the authors of the article and conducting 4 pilot interviews. The interview questions were repeated for all participants, but the order of the questions varied depending on their answers and the conditions of the interview.

**Table 1:** Checklist of interview guide question.

**No**.	**Questions**
1	What dilemma did you face after the coronavirus outbreak in Iran? Please explain.
2	What changes have taken place in your personal and family life during the days of quarantine and COVID-19? Explain.
3	Has there been a change in your physical or mental health? Explain.
4	What made you unable to follow health protocols (such as wearing a mask, keeping a social distance, etc.) during the COVID-19? Explain.
5	Has your relationship with your spouse changed these days (i.e., the quarantine and COVID-19) compared to the past? Explain.
6	Has your social relationship changed during this time? Explain.
7	Has anyone in your family had COVID-19? If yes, what were the most important problems you had? How was the treatment of the patient?
8	Has anyone in your family died of COVID-19? If yes, explain how the situation went and what problems you encountered after the patient death?
9	Were you satisfied with the performance of social and health organizations in relation to COVID-19 and the assistance they provided? If not, explain why.

The time and place of the interviews were determined by the participants. Most of the interviews took place in the afternoon, when participants had more time, and they were conducted in places such as participants' private homes, public places such as libraries, and parks. The average time of the interviews was 58 min, the shortest interview lasted 18 min and the longest interview lasted 70 min. During the interview, COVID-19 health protocols such as wearing masks and gloves, observing appropriate distance, etc. were observed by researchers and participants. Data collection lasted for 80 days from 9 April 2021 to 2 July 2021.

In this study, data collection continued until data saturation, which was achieved with 30 interviews. In qualitative research, saturation occurs when no new code or concept is obtained from interviews and the continuation of the interviews only leads to the formation of duplicated codes. However, the data were saturated in interview 26, due to the greater sensitivity of the researcher and the prevention of false saturation, four more interviews were done.

In this study, data analysis was performed by the first and third authors of the article using Graneheim and Lundman method (39). In the first stage, immediately after recording the first interview, on the same day, two researchers listened to the recorded interview file several times, which was recorded with the written consent of the participants, and typed it in a Word file. Then interview was reviewed several times to gain a general understanding of them. In the next step, all the texts of the interview were read carefully and wherever they thought it could be considered as a code, it was marked and the initial codes were extracted. In the next step, the codes that were similar to each other in terms of meaning and concept were placed in the same category, and then codes and categories were placed in a more comprehensive and complete category that was more abstract. Finally, the categories and subcategories were extracted.

Guba and Lincoln criteria were met to improve the quality of results (40). To increase the credibility of the research, the researchers observed the diversity in sampling and tried to have participants with as many different conditions as possible, such as age, place of residence, and so on. To gain Dependability, the research findings were provided to some participants (four people) to express their opinions. Also, at the end of each interview, the researcher briefly stated their general understanding of the participant statements to be approved by the participant. At the end, the analysis of the data and findings was sent to five researchers who had experience and skills in the field of qualitative research and the subject, who also confirmed the analysis and findings. To increase Confirmability, all the authors of the article were involved in analyzing and coding, and were present at the meetings and expressed their opinions. To increase transferability, a complete description of the whole research process was provided, and participants' quotes were given directly and in large numbers. The findings of the study were also shared with four people who had similar conditions to the participants in this study but were not present in our study, and they also confirmed that they had experiences similar to the experiences of the study participants.

In order to comply with the ethics code, all participants gave their written consent and were told that there was no compulsion to participate in the research and that they could leave the interview whenever they wanted. They were also briefed on the interviewer, the research process, and how the results were published, and were assured that their names would remain confidential when publishing the results.

## Results

Thirty people participated in this study, whose demographic characteristics are listed in Table 2. Also, from the data analysis, 143 primary codes, 12 subcategories and five main categories emerged from the data analysis, which are listed in Table 3.

**Table 2:** Demographic information of participants.

**Variable**	**Group**	**Frequency**
Age	Under 30	7
	30–50	17
	Over 50	6
Education	Illiterate	14
	Elementary	11
	Upper Elementary	5
Marital status	Married	25
	Single	5
Duration of stay in Iran	Under 5	3
	5–15	11
	Over 15	16
History of COVID-19 among first-degree relatives	Yes	22
	No	10
History of death caused by COVID-19 among first-degree relatives	Yes	13
	No	17

**Table 3:** Categories, subcategories and codes obtained from interviews with Afghan women.

**Categories**	**Subcategories**	**Codes**
Little knowledge and information	Limited access to information resources	Not having TV, not having internet, lack of literacy to read newspapers etc.
	Incomplete knowledge about COVID-19	Little knowledge about COVID-19, preventing it and treating it
Family challenges	Intensified experience of violence and conflict in the family	Verbal violence, psychological violence, physical violence, conflicts between family members
	Problems related to childbirth and pregnancy	Unwanted pregnancies, unsafe pregnancies, unsafe deliveries, abortion and lack of conditions for proper pregnancy
Socio-economic challenges	Exacerbation of economic problems	Unemployment of husband and children, expensiveness and inflation, the cost of buying hygienic products such as alcohol and masks
	High-risk living conditions	Not having proper bathrooms and toilets, overcrowded households, high-risk jobs (regarding COVID-19) of family members, and living in insecure neighborhoods regarding COVID-19
	Social isolation	Disconnection with friends and relatives, limited access to virtual communication, closed borders as an impediment to visit relatives in Afghanistan, separation from children
	Limited support of social and health organizations	Disregard for concessions for refugee treatment, poor financial and non-financial support, a lack of a proper mechanism for screening and testing among refugees and a lack of a proper mechanism for distributing health and livelihood support packages
Health issues	Problems related to treatment	Delay in seeing a doctor, fear of going to the hospital after infection, being forced to undergo treatment at home, self-medication (self-treatment), high cost of treatment, not having the right conditions for the patient's home quarantine, infecting all family members, Being rejected by some hospitals, inappropriate behavior of medical staff to Afghans, limited number of Afghan-related health centers
	Injustice in providing services and facilities	Difficult access of women to services, ignoring the needs of women, discrimination in providing services
Problems after the death of a COVID-19 patient	Burial challenges for immigrants	Ambiguity and fear of the burial conditions of a COVID-19 dead, lack of accountability of officials about the manner of burial, difficulty in transferring the body to Afghanistan, difficult conditions of burial in Iran, attempts to bury the body illegally
	Lack of funeral rites	Keeping the body at home, not holding burial according to their customs

### Little Knowledge and Information

This category consists of two sub-categories of limited access to information resources and incomplete knowledge about COVID-19. In fact, Afghan women had very limited knowledge about COVID-19, most of which was related to their limited access to information that prevented them from having proper information about various aspects of COVID-19.

#### Limited Access to Information Resources

Most Afghan women in Tehran were illiterate or had very low literacy, which made it difficult for them to access various information about COVID-19. There were many restrictions that limited their access to internet resources and even TV; this made their situation more difficult because they were even deprived of the necessary knowledge on how to take care of themselves against COVID-19.

“*I did not go out and we did not have a radio or television inside the house to follow the news about COVID-19.” (Participant No. 3)*

“*I remember early days they gave my wife a series of booklets about COVID-19, but I'm illiterate, I could not read, I just looked at its pictures.” (Participant No. 8)*

“*We do not have internet or a satellite at home to increase my information about COVID-19. Sometimes I ask my son how the condition is.” (Participant No. 21)*

#### Incomplete Knowledge About COVID-19

Many participants had very little knowledge of COVID-19 due to their limitations and this could endanger their health. Participants' knowledge about various aspects of COVID-19, such as the disease itself, prevention, treatment, etc., was very little, and since these women often took care of their husbands or children as nurses, this limited knowledge could make the situation much more dangerous.

“*I used the disposable mask several times, and sometimes I washed it until one day a friend of mine said that doing so endangers your health and is not right.” (Participant No. 12)*

“*I did not know much about what COVID-19 was, I just saw that everyone was afraid of it and talked about it, and because I had little information, I was very scared, too.” (Participant No. 19)*

“*I had not known anything about COVID-19 before my husband took it, I had only heard about COVID-19 from some of the neighbors.” (Participant No. 13)*

“*I had no idea at all how to protect myself against COVID-19.” (Participant No. 16)*

“*I just heard that we had to wash everything we got from the outside. I just heard that much about COVID-19.” (Participant No. 5)*.

“*When I got COVID-19, I thought I would get better by taking pills. I kept telling my husband to bring me the pill. I did not know that no pill could completely cure it.” (Participant No. 2)*

### Family Challenges

Another category is family challenges, which consists of two subcategories: intensified experience of violence and conflict in the family and problems related to childbirth and pregnancy. The COVID-19 outbreak affected all aspects of families' lives, and since Afghan families had more vulnerable situation as refugees, these effects were exacerbated, and tensions over the COVID-19 led to more family conflict and violence. Afghan women also faced problems with childbirth and pregnancy as public health declined.

#### Intensified Experience of Domestic Violence and Conflict

Afghan women are often exposed to violence due to their poor status and position and also traditional beliefs, but with the spread of the COVID-19 and the unemployment of their husbands and the increase in the family population, this violence was exacerbated. Also since the family's sons were normally vendors or working in small workshops with the closure of these activities, they were forced to stay at home, and this large population in small areas of the house automatically caused more tension and conflict.

“*When COVID-19 came and everywhere was closed, my husband was at home all the time and he was very upset about it. He was always arguing with me and the children.” (Participant No. 20)*

“*My husband is really upset that her income has decreased and he empties his anger on us. Sometimes he says bad things to me and sometimes she beats me.” (Participant No. 29)*

“*Before COVID-19 came, my son used to go out and work with their father or alone, but when COVID-19 came, everyone had to stay home and this caused everyone to have a fight each other all the time.” (Participant No. 6)*

“*Our house is 35 square meters. Earlier, everyone used to gather just to sleep, but on early days of COVID-19 when everywhere was closed, everyone was home from morning to night. This caused the children to fight and get on our nerves.” (Participant No. 15)*

#### Problems With Childbirth and Pregnancy

The outbreak of COVID-19 in Iran affected all aspects of Afghan women's lives, making their pregnancies riskier and made them confront dilemma such as unwanted pregnancies, unsafe pregnancies, unsafe deliveries and lack of conditions for proper pregnancy that could endanger the health of mothers and even their children.

“*I was taking birth control pills, but when COVID-19 came, I did not go out anymore and my husband could not buy the pill because of his shyness, which led to my pregnancy.” (Participant No. 4)*

“*I was pregnant during COVID-19 outbreak. I got very bothered. It was the hardest pregnancy of my life. I could not go to any doctor due to fear of COVID-19. I did not know at all whether my child was healthy or not. I had a lot of stress till my baby was born.” (Participant No. 17)*

“*I was pregnant last year. For fear of COVID-19, I did not go to the doctor at all. When I went, I was said that my baby had a problem. I aborted it; if I went to doctor sooner, maybe I it wouldn't be necessary to have abortion” (Participant No.)*

“*When I wanted to give birth to my baby, I had to give birth at home. It was very difficult. Our neighbor, who was a midwife, came over to help me. I was very scared.” (Participant No. 30)*

“*After COVID-19 came, I got pregnant, but the conditions were not good at all. Our income had decreased and we could not have a proper diet like before. Sometimes we did not eat fruit even once a month, and we could not eat meat that was so expensive that we could not afford it at all.” (Participant No. 17)*

### Socio-Economic Challenges

Afghan women as a weak section of Iranian society in the face COVID-19 due to the limitations as well as their fragile economic conditions and the discrimination that is applied to them confront many social and economic challenges; this can delay the process of adapting to face COVID-19. This category consists of the sub-categories of exacerbation of economic problems, high-risk living conditions, social isolation and limited support of social and health organizations.

#### Exacerbation of Economic Problems

Along with the outbreak of COVID-19 in Iran and the closure of many businesses, Afghans suffered a great deal of pressure. As some of the children of these Afghans were vendors in the streets and with the spread of COVID-19 no one bought from them, this economic pressure increased and on the other hand, the purchase of hygienic products imposed a lot of costs on them.

“*My husband was a vendor. With the arrival of COVID-19, no one bought anything from him anymore, and this put us under a lot of pressure.” (Participant No. 1)*

“*In these two years, everything has become more expensive, you can't buy anything at all. Our life used to be better, but now it has gotten much worse.” (Participant No. 11)*

“*In the beginning, we could not find alcohol and a mask at all, but then it got better, but it is very expensive for us, so we have to use a disposable mask for several days, or even when we buy alcohol, we pour half more water to make it more so that we can use it for a longer time.” (Participant No. 16)*

“*We are under a lot of pressure. Our income has decreased. My sons used to work outside as vendors. Now all of them are unemployed.” (Participant No. 24)*

#### High-Risk Living Conditions

Afghans live in very difficult conditions in Iran, and because most of them are illegal immigrants, they are forced to work in low-income jobs and in poor conditions, which complicates their living conditions. Also, most of these refugees live in densely populated slums, and because these Afghan families themselves are large, sometimes 13 people have to live together in a 40-square-meter house, which can make conditions for quarantine and prevention COVID-19 difficult and impossible. Most Afghan women stated that they lived in places where they faced dilemma such as not having proper bathrooms and toilets, overcrowded households, high-risk jobs (regarding COVID-19) of family members, and living in insecure neighborhoods regarding COVID-19. These conditions could increase the risk of getting COVID-19, and if they got the disease, their lives would be more at risk due to the lack of proper treatment.

“*We are ten people living in a 40-square-meter house. There are several other families living in our yard.” (Participant No. 18)*

“*Sometimes we are told to be careful about Covide-19, but how can it be observed when so many people live in this small environment with a shared bathroom.” (Participant No. 10)*

“*There is only one bathroom in this whole house where there are 3 families. Feeling ashamed, we women often do not go to the bathroom.” (Participant No. 12)*

“*Our alley is so crowded in the evenings when the children go out. It is so crowded that it makes no sense to wear a mask at all.” (Participant No. 27)*

“*My husband and children are vendors most of the time and are in contact with a lot of people every day; it's inevitable, they have to go so that they can make money for the family.” (Participant No. 21)*

#### Social Isolation

With the outbreak of COVID-19 in Iran, travel was banned in many cities, as well as restrictions on travel to other countries such as Afghanistan. Most participants said closing borders caused some problems for them such as cutting the contact with relatives and friends in Afghanistan and separation from children. Also, because Afghan women were illiterate and had very limited access to the Internet, they could not use social media to communicate like other people, which exacerbated their social isolation.

“*Before COVID-19, I visited my sister at least once or twice a week, but after COVID-19 I was afraid to go; I'm home most of the time.” (Participant No. 24)*

“*After COVID-19, I stayed at home for a long time and had no contact with anyone so that I feel depressed.” (Participant No. 7)*

“*In previous years, we went to Afghanistan at least once or twice a year, but after COVID-19, we did not go; the borders are closed. My son lives in Afghanistan. I miss him very much. I cry for him every day.” (Participant No. 17)*

“*Some relatives communicate via the Internet, but I do not have a phone to have a video call.” (Participant No. 4)*

“*My husband sometimes talks to my son in Kabul at work, but we do not have internet at home. I have not seen or talked to him for more than a year.” (Participant No. 17)*

“*I miss my family very much, but we have neither phone to call nor internet.” (Participant No. 9)*

#### Limited Support of Social and Health Organizations

With the COVID-19 outbreak in Iran, Afghan refugees initially faced many problems and restrictions, but the Iranian government provided solutions such as the COVID-19 screening plan for foreigners, preventive services and a special hospital for foreigners, distributing disinfectants, awareness-raising measures and self-care guidelines. However, these solutions did not completely solve the refugees' problems; as most participants expressed they have been faced with problems such as disregard for concessions for refugee treatment, poor financial and non-financial support, a lack of a proper mechanism for screening and testing among refugees and a lack of a proper mechanism for distributing health and livelihood support packages.

“*Some centers provided some assistance during the COVID-19 outbreak, but it was very small.” (Participant No. 3)*

“*During the whole COVID-19 period, they only helped us twice; one time they brought disinfectants and masks, the other time they brought food packs, but it was very little and it ran out soon.” (Participant No. 8)*

“*Sometimes they told us that we could go to a certain place to have a COVID-19 test, but it was not at all clear what was going on. They were very undisciplined; sometimes they didn't care us.” (Participant No. 20)*

### Health Issues

This category consists of subcategories of problems related to treatment and injustice in providing services and facilities. The outbreak of COVID-19 created many health issues throughout Iran, but these problems were greater in the refugee population due to political and social restrictions.

#### Treatment Problems

Many participants stated that if they got COVID-19 they would have many issues that could delay their treatment and possibly would cause them to die. Some of their challenges were related to illegal immigration, so due to the fear of deportation to Afghanistan they tried to avoid going to the hospital as much as possible, and another part was related to the high cost of treatment for Afghans who could not afford it due to lack of insurance. Some other participants also pointed out the inappropriate behavior of the medical staff and the lack of suitable conditions for quarantine and the lack of hospitals.

“*I knew I had COVID-19 but I was afraid of going to the doctor because I am an illegal immigrant in Iran. My husband wanted to take me to the doctor several times, but I did not go myself because of fear.” (Participant No. 28)*

“*When my daughter got COVID-19, we took her to several hospitals, but as soon as they saw that we were Afghans, they rejected us.” (Participant No. 6)*

“*I had to be treated at home, so it was very difficult for us, because our house was small and I could not be quarantined at all, we all got COVID-19.” (Participant No. 13)*

“*For two nights in the hospital, we paid as much as a month's salary. Even though I was in a bad situation, we returned home. During the two nights we were in the hospital, some of the nurses treated me very badly. When they found out I was Afghan, they treated me badly.” (Participant No. 14)*

“*After I found out, I got COVID-19, I stayed at home and did self-treatment. I drank herbal tea and ate hot things all the time. It took me a long time to get well, but I had no choice.” (Participant No. 22)*

“*There were very few places for testing and treating Afghan patients, however many were even unaware of this.” (Participant No. 7)*

“*One of our neighbors got COVID-19, and we were very upset because we had a shared bathroom. The infection of this person caused most of the people living in the house to get COVID-19.” (Participant No. 10)*

#### Injustice in Providing Services and Facilities

Most of the participants stated that there was injustice in providing services and facilities because on the one hand the needs of women were ignored in providing the services and on the other hand since refugee women are mostly at home and have less access to the outside world they are deprived of receiving many of the services provided. Also, in some cases, some families received more services due to their relations, while some other families were completely deprived.

“*I, who never went out after COVID-19, never had anyone come to me to ask if you needed anything, and when they want to give something to us, my husband or son takes it.” (Participant No. 29)*

“*Once or twice I heard that they wanted to give us some things, but because my husband was not in Iran, I felt embarrassed and didn't go to take it.” (Participant No. 17)*

“*Some families who have contact with the authorities received food and hygiene items several times, but we only received them once, but our neighbor did not receive them even once.” (Participant No. 1)*

“*It is not clear how they distribute, they help some people several times, but they do not help others at all.” (Participant No. 21)*

### Problems After the Death of a COVID-19 Patient

This category consists of two sub-categories of burial challenges for immigrants and lack of funeral rites.

#### Burial Challenges for Immigrants

Afghan Refugees, in addition to having difficulty in preventing and treating patients with COVID-19, had issues after the death of these COVID-19 patients because they did not know how to bury their bodies because of their illegal residency. Sometimes they tried to bury the body illegally. They also faced other issues such as ambiguity and fear of the burial conditions of a COVID-19 dead, lack of accountability of officials about the manner of burial and the difficulty of transferring the body to Afghanistan, and the difficult burial conditions in Iran.

“*When my husband died because of Covod-19, we did not know what to do because my husband was illegal in Iran.” (Participant No. 14)*

“*My daughter died at home because of COVID-19. Her body was at home for a day or two. We did not know what to do for her burial. We called several places, but no one answered us correctly.” (Participant No. 24)*

“*In his will, our relative wanted to be buried in Afghanistan, but when he died of COVID-19 and we wanted to transfer his body to Afghanistan, we suffered a lot to cross the border and spent a lot of money. Finally, we could not take him to Afghanistan. We had to bury him in Iran.” (Participant No. 8)*

#### Lack of Funeral Rites

After the death of COVID-19 among Afghan refugees due to the conditions and restrictions that existed, they could not hold rites for them as usual and this issue made them annoyed. Sometimes due to issues and also to be able to bury the body according to their customs, they kept the dead body at home for several days, which could endanger the health of the rest of the family.

“*We Afghans have a series of special ceremonies after the death, but these ceremonies could not be held after COVID-19, and it was annoying.” (Participant No. 25)*

“*In general, after COVID-19, the conditions for burying corpses in Iran had become more difficult, and especially for us Afghans, it had become more difficult. Many times family members were not allowed to be beside the body at the funeral while we used to be beside the body at the funeral.” (Participant No. 2)*

“*When my husband died, we had to keep her body at home for a few days.” (Participant No. 7)*

## Discussion

The present study aimed to explore the challenges of Afghan refugee women facing COVID-19 in Iran with a qualitative approach. The results showed that Afghan women confront many dilemma in the face of COVID-19, both in the prevention and treatment of the disease, and even after the death of a COVID-19 patient, which can endanger their health and that of their families. Previous research has also shown that in the face of natural and unnatural disasters, refugees are among the vulnerable groups who are exposed to all kinds of harms (28, 41, 42). Women also have more limited access to services due to gender, cultural contexts, etc. and have to deal with more issues during natural and health disasters (20, 43).

Limited access to information resources and incomplete knowledge about COVID-19 was one of the interesting findings of this study. A study conducted by Fouad et al. among Syrian refugees in Lebanon showed that refugees have little knowledge of COVID-19 (33). Yücel also stated in his research that during the COVID-19 pandemic, Syrian refugees in Turkey are ignored by the media and this issue further aggravates inequalities (44). In fact, Afghan women were deprived of access to information due to insufficient literacy to study and lack of information resources such as television, etc., and this deprivation made them very vulnerable in the face of COVID-19 because it deprived them of sufficient knowledge and awareness so that in many cases the participants had incomplete and little information about COVID-19 and how to prevent and treat it. Therefore, it is necessary to educate women and also to provide conditions for them to have access to various information about COVID-19.

Intensified experience of violence and conflict in the family was another research finding that is consistent with most research on COVID-19 (45–47). A study by Mutambara et al. among refugee women in South Africa also reported an increase in violence against women during the COVID-19 (35). The COVID-19 outbreak has affected all aspects of Afghans life and put many restrictions on their lives, forcing family members, who used to be outside for longer hours and used to return home only to sleep, to stay home all day. This issue has affected the lifestyle of these families. These changes cause stress and anxiety to the family members, and because their income level has also decreased and also due to the limited space of the house, it may lead to aggression and violence against women. It can be said that women are also under more pressure due to taking care of family members, child care and home education (48).

Problems related to childbirth and pregnancy were another new finding in this study. Access to contraceptives often becomes more difficult in critical situations in countries where there are cultural taboos on sex, and this can lead to unwanted pregnancies. Also, more presence of men at home and limited access to contraceptives caused some women to experience unwanted pregnancies, and due to limited access to health services, they experienced unsafe pregnancies and even unsafe deliveries which endangered the health of women and their infants. Of course, economic pressures and change in family circumstances could also increase these risks. In the study of Kumar et al., the increase of unsafe abortion has been reported as one of the consequences of COVID-19 (49). Unwanted and unsafe pregnancy has also been reported as another COVID-19 consequence in several studies (50–52). Therefore, it is necessary to provide conditions in which Afghan women can easily access contraceptives. In this regard, female facilitators familiar with the culture and traditions of Afghan women can also be used. It is also possible to provide them with healthier pregnancies and deliveries through virtual training and the provision of appropriate grounds for more access to health services.

Another finding of the study was the exacerbation of economic challenges that is in line with previous studies conducted in different countries of the world (1, 7, 53). With the outbreak of COVID-19, almost all businesses were partially or completely shut down, and businesses that did not shut down fell into recession due to restrictions, causing losses to workers and employers. But what made the situation of Afghan refugees in Iran worse was that most of these workers were engaged in jobs that lacked insurance and a specific compensation mechanism due to their illegal residency. Most of the refugees were engaged in jobs such as vendor, which the outbreak of COVID-19 made people rarely willing to buy anything from them; this hurt the economy of Afghan families. There was also the addition of other expenses, such as the purchase of masks and disinfectants to prevent COVID-19, and on the other hand, the economic situation of the whole country was not very good due to the sanctions against Iran.

High-risk living conditions were another significant finding in this study. Most studies have reported that refugees are recognized as one of the most vulnerable groups in the COVID-19 era due to inadequate housing and lack of access to health facilities, etc. (27–29, 42). In the study of Fouad et al., the large population of refugee families and inadequate housing have been reported as one of the risk factors for COVID-19 (33). In the present study, most of the participants had to live in small houses as well as high-density neighborhoods while having large families, which could make the conditions related to COVID-19 prevention difficult or even impossible. Having high-risk jobs that exposed Afghans to large crowds all day was also another problem for Afghan refugees. In fact, it can be said that Afghan refugees are forced to live in insecure neighborhoods and have high-risk jobs due to their social and economic situation, which puts their health more at risk than other members of society.

Social isolation was another finding of the study that was reported in many other studies due to conditions caused by COVID-19 and quarantine and restrictions, and it seemed natural (54–56). But what was new was that, unlike other studies in which people turned to virtual communication and saw it as a substitute for real communication (30, 57), the women in this study due to the limited access to the Internet and cell phone, were also deprived of virtual communication, and this deprivation intensified their social isolation.

Limited support from social and health organizations was another concerning finding in this study. Other studies have cited limited access to social and health services as one of the main refugee issues facing COVID-19 (58). Part of this limited support was related to the economic and social situation in Iran, which had many problems due to US government sanctions, and most of it was related to the illegality of Afghan refugees because there were no accurate statistics on their presence and their being unidentifiable made it difficult to get support of social and health organizations.

Another finding of this study was the dilemma that Afghan women faced when getting COVID-19. After getting COVID-19, Afghan refugees tried to go to doctors and medical centers as late as possible due to fear, and since they did not have proper quarantine conditions, the situation became more difficult for them and could jeopardize the health of all family members. This finding was in agreement with the research of Salmani et al. (37). Of course, in some cases, the bad behavior of the medical staff and the high cost of treatment were also effective, because due to their low income and lack of insurance, the cost of hospitalization was very high for them and they tried as much as possible to do treatment at home.

Problems after the death of a COVID-19 patient was another problem faced by Afghan refugees. COVID-19 has caused a great deal of change in burial conditions around the world, and Iran, like other countries, has introduced laws and restrictions on funerals to prevent more people from becoming infected. But what made the situation of Afghan refugees more difficult was that they did not know how the body would be buried under the new circumstances, and the authorities did not provide adequate training and knowledge to the refugees. Also, in cases where they had an illegal presence in Iran, the situation became more complicated, so that some families kept the corpse at home for several days and even tried to bury it illegally, which due to the impossibility of observing burial health protocols could make the situation of families more critical. Therefore, it can be said that it is necessary to inform the refugees about how to bury a COVID-19 dead and also to provide conditions so that even those who are illegally present in Iran can easily bury their COVID-19 dead.

Injustice in providing services and facilities was another finding of the study that added to previous research that women, in addition to being discriminated against as refugees, are discriminated against and treated unfairly within their own class as women. Refugee women are more isolated than men. This limits their access to services, and since there is no knowing and understanding of their situation, their needs are ignored, making it even more difficult for them. It should be noted that among the refugees, some families are given more attention and support for reasons such as longer stay in Iran and better communication with health distribution officials, and this issue itself causes more discrimination.

## Limitations and Strengths of Research

This study is one of the few studies that qualitatively examines the experiences and problems of refugee women in Iran and the world during the COVID-19, which can provide significant information to the audience and social and health organizations to reduce these problems. However, there were some limitations in conducting this study. Due to the illegality of the presence of most participants in Iran, it was difficult to access and identify them. Using snowball sampling method and getting help from local facilitators, researchers were able to overcome this limitation. Another limitation was gaining the participants' trust to consent to participate in the research, which was gained by describing the research process and assuring them that their interview was recorded only for the purposes of the research and was not provided to any organization. Another limitation of the research was the interview location, it was not possible for many women to take part in the interview outdoors and it was crowded indoors, and participants may not be able to easily express their experiences. Interviews should be conducted when no one is home or if another person was at home they were politely asked to leave the interview place. Another major limitation was that due to the COVID-19 spread, some participants were afraid of face-to-face interviews, which the researchers initially tried to gain their consent by reassuring them to follow health protocols such as wearing masks and gloves, as well as keeping the appropriate distance. And then in case of dissatisfaction for in-person interview, telephone interview was used.

## Conclusion

The results showed that Afghan women in Iran during the COVID-19 period confronted many problems such as little knowledge and information, family challenges, socio-economic challenges, health issues, and problems after the death of a COVID-19 patient that could endanger their health, and even lead to their death. Therefore, in the first stage, it is necessary to raise awareness on various aspect of COVID-19, such as prevention, treatment, etc., and in the later stages, governmental and non-governmental organizations should provide more appropriate social and health services for Afghan refugees. And in providing these services and facilities, women should be given more attention as the most vulnerable group of Afghan refugees so that they can access these services more easily and without any restrictions. Also, providing more financial assistance, as well as increasing the number of medical and health centers for Afghan refugees, and making the treatment and prevention process less expensive, can increase their access so that they can better protect themselves against the disease.

## Data Availability Statement

The datasets used and/or analyzed during the current study are available from the corresponding author on reasonable request.

## Ethics Statement

Ethical approval was obtained from the Iran University of Medical Sciences (IR.IUMS.REC.1399.500). Written consent was obtained from all participants. All procedures performed in studies involving human participants were in accordance with the ethical standards of the institutional and/or national research committee and with the 1964 Helsinki declaration and its later amendments or comparable ethical standards.

## Author Contributions

AD and HE contributed to designing the study. JY and AD collected the data and analyzed it by SI and JY. The final report and article were written by SI, JY, and HE. All authors participated and approved the study design and read and approved the final manuscript.

## Conflict of Interest

The authors declare that the research was conducted in the absence of any commercial or financial relationships that could be construed as a potential conflict of interest.

## Publisher's Note

All claims expressed in this article are solely those of the authors and do not necessarily represent those of their affiliated organizations, or those of the publisher, the editors and the reviewers. Any product that may be evaluated in this article, or claim that may be made by its manufacturer, is not guaranteed or endorsed by the publisher.

## Footnotes

^1^Available online at: www.worldometers.info, https://www.worldometers.info/coronavirus/ (2021).
